# An Image-Based Framework for Measuring the Prestress Level in CFRP Laminates: Experimental Validation

**DOI:** 10.3390/ma16051813

**Published:** 2023-02-22

**Authors:** Jónatas Valença, Cláudia Ferreira, André G. Araújo, Eduardo Júlio

**Affiliations:** 1 CERIS, IST-ID, University of Lisbon, 1049-003 Lisboa, Portugal; 2 Institute of Systems and Robotics, University of Coimbra, 3030-290 Coimbra, Portugal; 3 Ingeniarius, Lda, 4445-147 Porto, Portugal; 4 CERIS, IST, University of Lisbon, 1049-001 Lisboa, Portugal

**Keywords:** machine learning, deep learning, computer vision, CFRP laminates, strengthening RC, strain monitoring

## Abstract

Image-based methods have been applied to support structural monitoring, product and material testing, and quality control. Lately, deep learning for compute vision is the trend, requiring large and labelled datasets for training and validation, which is often difficult to obtain. The use of synthetic datasets is often applying for data augmentation in different fields. An architecture based on computer vision was proposed to measure strain during prestressing in CFRP laminates. The contact-free architecture was fed by synthetic image datasets and benchmarked for machine learning and deep learning algorithms. The use of these data for monitoring real applications will contribute towards spreading the new monitoring approach, increasing the quality control of the material and application procedure, as well as structural safety. In this paper, the best architecture was validated during experimental tests, to evaluate the performance in real applications from pre-trained synthetic data. The results demonstrate that the architecture implemented enables estimating intermediate strain values, i.e., within the range of training dataset values, but it does not allow for estimating strain values outside those range. The architecture allowed for estimating the strain in real images with an error ∼0.5%, higher than that obtained with synthetic images. Finally, it was not possible to estimate the strain in real cases from the training performed with the synthetic dataset.

## 1. Introduction

Image-based methods for civil engineering applications have been developed in the last 20 years. Several methods were developed in the scope of Structural Health Monitoring (SHM), product and material testing, and quality control. Applications based on photogrammetry and image processing are used to support inspection and monitoring of infrastructures, allowing for computing displacement and deformation fields [1,2,3,4], curvatures and rotations [5], and mapping and characterizing anomalies [6,7]. Lately, machine learning and deep learning for compute vision is the trend followed, taking advantage of all the technology available [8,9,10]. Its application to damage analysis and reliability assessment is promising and has several advantages [11,12,13].

The deep learning applications require large and labelled datasets for training and validation. Furthermore, it is often not possible to generalize and apply outside the limits of validation of the training dataset. The data augmentation using synthetic datasets is a possible solution to add knowledge to the networks developed. One of the most applied and successful artificial neural networks (ANNs) for structured regression problems is the ResNet, a deep neural network with hundreds of layers and skip connections between layers [14]. This Convolutional Neural Network (CNN) is broadly applied and trained with synthetic data in several areas of knowledge. However, there is no consensus about the size of the dataset as well as the reliability of using synthetic images in training as data augmentation. Ward et al. [15] use ResNet34 for ships classification, by training the neural network with synthetic and real images, and compare the performance with classical object recognition methods. The dataset of real images is composed of 200 images while the synthetic dataset is composed of 200k images. To understand the effects of data dispersion on different object recognition approaches, these authors tested five ratios for data splitting, with 20% of the training dataset used for validation. Many developments have taken place in the field of medicine. Lei et al. [16] use ResNet34 to diagnose congenital heart disease in a fetus through the analysis of computed tomography images. The original dataset is composed of 1729 images, in which 1371 images of normal hearts and 358 of hearts with anomalies. To balance the dataset, the last group was duplicated twice, and the final dataset is composed of 2445 images: 1371 images of normal hearts and 1074 of hearts with anomalies. The test dataset is composed of 200 images of normal hearts and 200 of hearts with anomalies. An accuracy of 80.7% was reached at the test stage.

Al-Moosawi and Khudeyer [17], among other methodologies, implement ResNet34 for the diagnosis of diabetic retinopathy. The dataset consists of 4075 images, and the distribution of images is not uniform across the different stages of the disease. The percentages of the training, validation, and test dataset are, respectively, 67.5%, 22.5% and 10%, and an accuracy of 94.9% was calculated. Yadav et al. [18] use ResNet34 and ResNet50 for the detection of patients infected with COVID-19 pneumonia from chest X-rays. The dataset consists of 2481 images, with 80% of the images being used for training and 20% for testing. The results reveal an accuracy of 94.4% for ResNet34, and 96.4% for ResNet50. Other fields, such as Biology, are also using these type of approaches. Pavel et al. [19] use ResNet34 to identify diseases in plants from images of their leaves. The dataset is composed of 7600 images (200 images for each category). In this case, 6080 images (80%) were used for training and 1520 images (20%) for validation. The model was trained in 15 epochs and reach 97.0% of accuracy. Gao et al. [20] use ResNet34 combined with transfer learning to detect defects in wood. Before data augmentation, the dataset consisted of 448 spruce defects, split in a ratio of 6:2:2 for the training, validation and test datasets, respectively. After increasing the data, the dataset stays with 3136 images, 1885 images for training, 636 images for validation and 615 images for testing. The model uses 300 epochs and hit 98.7% accuracy at the test stage.

The use of Carbon Fibre Reinforced Polymers (CFRP) has been successfully applied in several areas including repair and rehabilitation of reinforced concrete (RC) structures [21,22]. The technique allows a significant improvement in the flexural and shear strength of concrete members. One of the most used methods consists of externally bonded reinforcement (EBR) of concrete members with CFRP laminates [23]. For large span elements, the application of prestressed CFRP laminates is an advantageous solution for both ultimate and service limit states [24]. In these cases, the level of prestress applied can be evaluated directly by measuring the strain in the laminates. This can be achieved using strain gauges [25,26,27,28,29,30] or fiber optic sensors [31,32]. Both cases require instrumentation of the structures, becoming time-consuming and laborious, and thus just applied in special cases. A contact-free architecture for a vision-based system was proposed and benchmarked by the authors [33]. The architecture was analysed with a dataset of synthetic images and testing machine learning and deep learning algorithms. A data augmentation based on the application of filters to mimic real scenarios was also performed. ResNet34 provided the most accurate results, reaching a root mean square error (RMSE) of lower than 0.1% for strain prediction.

In this paper, the developed architecture is validated through the application in an experimental test. The main goal is to evaluate the application of the architecture in real images of CFRP laminates during prestress application. Specifically, the aim is to assess whether the architecture developed allows for estimating:

- Intermediate strain values within the range of training dataset values;
- Strain values greater than the range of training dataset values;
- Strain in real images with an accuracy identical to that obtained with synthetic images;
- Strain in real images from training synthetic images.

The analysis and responses to these specific objectives will allow for defining the limits of validity of the proposed architecture for real applications. This represents an important contribution to the dissemination of the new monitoring approach, which will increase the number of conveniently monitored reinforcement applications, promoting quality of execution and greater structural safety in the construction sector.

## 2. Methodology

**Figure 1:**
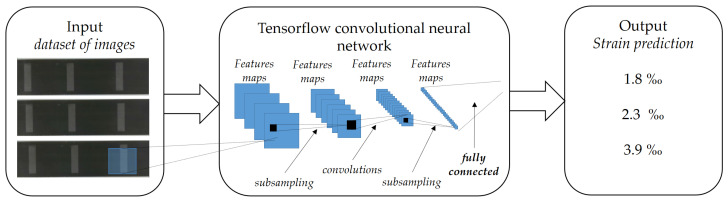
Deep learning architecture implemented.

The architecture based on computer vision for strain monitoring of CFRP laminates was tested for machine learning and deep learning. The results indicate deep learning with regression as the better solution for the problem [33]. The solution was implemented with ResNet34 as a backbone network and tested with the synthetic images dataset (Figure 1). ResNet is a deep neural network that considers over one hundred layers without vanishing gradient problems for training [34,35] and uses the skip connection technique. The last layer’s activation function is replaced by a linear activation function, taking into account the mean square error loss and, for ResNet34, 34 layers. These characteristics lead to a more flexible Convolutional Neural Network (CNN) structure. The main block of ResNet34, presented in the center of Figure 1, is composed by:

- Convolutional layers, to extract features from the images;
- Batch normalizations (BNs), to accelerate training and provide regularization;
- Rectified linear unit (ReLU) activation function, to control the exponential growth in computation; and
- Shortcut, for skip layers in the input of the next step.

The deep learning algorithms are integrated using open source platforms, namely TensorFlow and Scikit-Learn [33].

**Figure 2:**
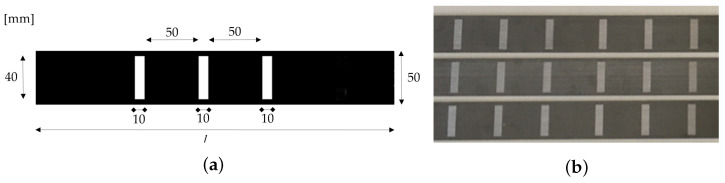
Pattern designed on CFRP laminates: (**a**) synthetic images and dimensions of the pattern; (**b**) image of real laminates.

A pattern of three strips, each with 10 mm × 40 mm spaced 50 mm apart, was considered on the surface of the laminates for monitoring proposes. The architecture is fed by images with different levels of strain, for training, validation and testing (Figure 2a). In the case of synthetic images, aiming to mimic real case scenarios, a set of filters were applied, and the dataset was built following the recommendation of [33]: (i) Gaussian noise, to simulate the effect of thermal noise on the sensor [36]; (ii) salt noise, to reproduce overexposed bright pixels [37]; (iii) pepper noise, to underexposed dark pixels [37]; (iv) salt and pepper noise, combining the last two [37]; (v) speckle noise, to mimic the interference phenomenon due to surfaces roughness [38]; and (vi) Poisson noise, representing the electromagnetic waves at infrared waves [39]. The pattern defined was laser printed in CFRP laminates for measuring real cases (Figure 2b).

## 3. Experimental Validation

### 3.1. Set-Up and Material

**Figure 3:**
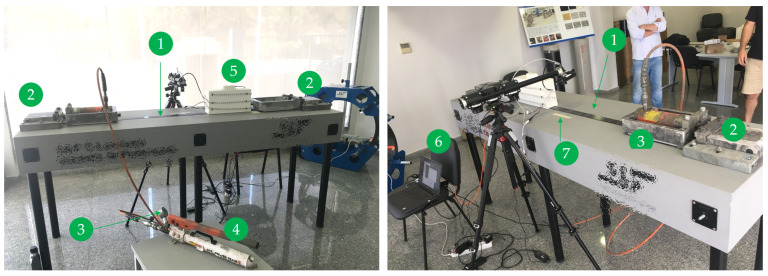
Experimental set-up overview.

This section presents the experimental test conducted to validate the architecture implemented. Figure 3 shows an overview of the entire set-up mounted to apply and monitor the application of prestress in the CFRP laminates that comprises the following:

1. CFRP laminate;
2. Anchor plates system;
3. Hydraulic jacks system;
4. Pressure manometer;
5. RealSense D435 Camera;
6. Control computer;
7. Millimeter ruler.

**Figure 4:**
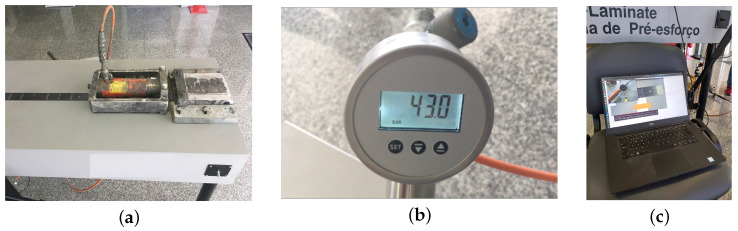
Experimental test set-up: (**a**) hydraulic jack for prestress application; (**b**) manometer; (**c**) control computer for strain monitoring.

The CFRP laminates are produced with unidirectional fiber reinforcement in the direction of the laminate and embedded in a polymer resin. The laminate tested is available in 150 m rolls, and is 50 mm wide and 1.4 mm thick. In terms of the mechanical characteristics, the laminate has a modulus of elasticity of approximately 170 kN/mm^2^, and the prestress usually applied leads to strains of between 6% and 8%. The laminates are anchored in a steel reinforced table, and a hydraulic jack system is used to apply a unidirectional deformation along the laminate axis, by a manual applied pressure (Figure 4). A millimeter ruler placed in the center of the laminate also enables measuring the displacements during the application of the prestress. For image acquisition, a RealSense D435 Camera, mounted in a specific support box to capture images during prestress application at a predefined distance and with the same light conditions, is used. All of the data acquisition, from the camera to the manometer, is synchronized with the control computer hour (Figure 4c).

### 3.2. Data Acquisition and Preparation

The strain on the laminate can be estimated from the pressure in the manometer by:

(1) $$\varepsilon =\frac{Am\times P}{Al\times El}$$

where Am, in mm^2^, is the area of the hydraulic jack piston (3882 mm^2^ for this jack); *P*, in MPa, the pressure observed on the manometer; Al, in mm^2^, the area of the laminate (for the present case study, 70 mm^2^); and El, in GPa, the modulus of elasticity of the laminate (170 GPa for this laminate).

The camera has a sensor size of 1751 px × 1493 px and a focal length of 1.93 mm. This leads to acquiring images with 330 mm of laminate, identical to the synthetic images produced. Furthermore, the camera was programmed for an acquisition frequency of 2 Hz, in order to create the dataset for offline testing.

**Figure 5:**
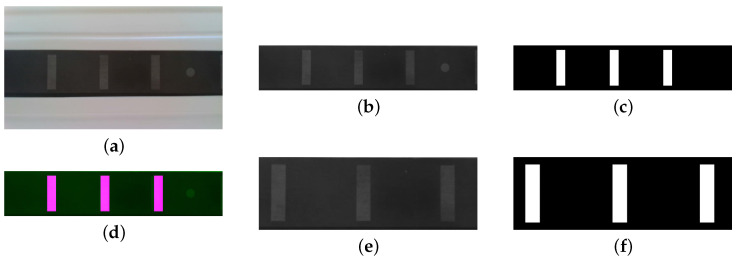
Dataset preparation: (**a**) real image acquired; (**b**) real image cropped; (**c**) synthetic image; (**d**) real and synthetic image overlap; (**e**) final real image; (**f**) final synthetic image.

The synthetic images were generated to have the same resolution as the real images and be in accordance with Section 2. Figure 5a shows an acquired real image, with a field of view (FOV) that leads to an image length of 24.5 cm or 1920 px. Then, the real images are cropped to select only the regions of interest (Figure 5b), and the synthetic images are computed to match this image (Figure 5c). This can be confirmed by overlapping both images at stage 0, i.e, with no strain applied, as in Figure 5d. To optimize the computational cost, the final images used in the datasets are cropped, taking into account that the central stripe matches the image centre (Figure 5e,f).

### 3.3. Training, Validation and Testing

**Figure 6:**
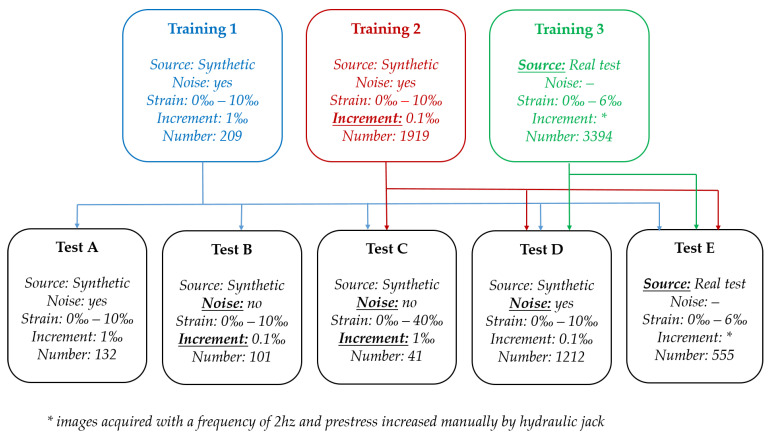
Schema of the analysed datasets.

The datasets were built to answer the specific goals set in Section 1. Thus, three training datasets and five testing datasets were defined, as in Figure 6. The level of strain imposed is within the limits of the material for real case tests (6%), and above this limit in the case of simulation with synthetic images (10%). In the following sections, all the details of each of those datasets are presented and described.

#### 3.3.1. Training and Validation Datasets

The three training datasets referred to in Figure 6 are described below.

- **Training 1**—the first training dataset is solely composed of synthetic images, with a strain range from 0% to 10% with an incremental step of 1%. The dataset has 209 images:
  - 11 synthetic images without noise (1 image for each strain value);
  - 33 synthetic images with Gaussian noise (3 images for each strain value);
  - 33 synthetic images with Pepper noise (3 images for each strain value);
  - 33 synthetic images with Poisson noise (3 images for each strain value);
  - 33 synthetic images with Salt noise (3 images for each strain value);
  - 33 synthetic images with Salt and Pepper noise (3 images for each strain value);
  - 33 synthetic images with Speckle noise (3 images for each strain value);
- **Training 2**—the second training dataset is also composed of synthetic images, with a strain range from 0% to 10%. To make the dataset more realistic and decrease the error for strain prediction, the step between strain was reduced from 1% to 0.1%. The dataset consists of 1919 images:
  - 101 synthetic images without noise (1 image for each strain value);
  - 303 synthetic images with Gaussian noise (3 images for each strain value);
  - 303 synthetic images with Pepper noise (3 images for each strain value);
  - 303 synthetic images with Poisson noise (3 images for each strain value);
  - 303 synthetic images with Salt noise (3 images for each strain value);
  - 303 synthetic images with Salt and Pepper noise (3 images for each strain value);
  - 303 synthetic images with Speckle noise (3 images for each strain value);
- **Training 3**—the dataset is only composed by real images acquired for strain values between 0% and 6%. The images were acquired with a frequency of 2 Hz, and a dataset with 3394 images was produced. It is also important to mention that the real laminate behaved according to what was expected for the levels of strain imposed.

Furthermore, it should be mentioned that 20% of the images from each training dataset were used to build the validation dataset. This division of the datasets was performed randomly.

#### 3.3.2. Test Datasets

The five test datasets (Figure 6) were built as below.

- **Test A**—synthetic images deformed for a strain range between 0% and 10% with a step of 1%. More specifically, 132 synthetic images:
  - 22 synthetic images with Gaussian noise (2 images for each strain value);
  - 22 synthetic images with Pepper noise (2 images for each strain value);
  - 22 synthetic images with Poisson noise (2 images for each strain value);
  - 22 synthetic images with Salt noise (2 images for each strain value);
  - 22 synthetic images with Salt and Pepper noise (2 images for each strain value);
  - 22 synthetic images with Speckle noise (2 images for each strain value);
- **Test B**—synthetic image without noise deformed for a strain between 0% and 10% with a step of 0.1%, with 101 images, one for each strain value;
- **Test C**—synthetic image without noise deformed for a strain between 0% and 40% with a step of 1%, in a total of 41 images;
- **Test D**—synthetic image with noise deformed for a strain between 0% and 10% with a step of 0.1%, in a total of 1212 images, two images for each strain value and for each type of noise;
- **Test E**—real images with strain values between 0% and 6%, in a total of 555 images.

## 4. Analysis of Results

**Figure 7:**
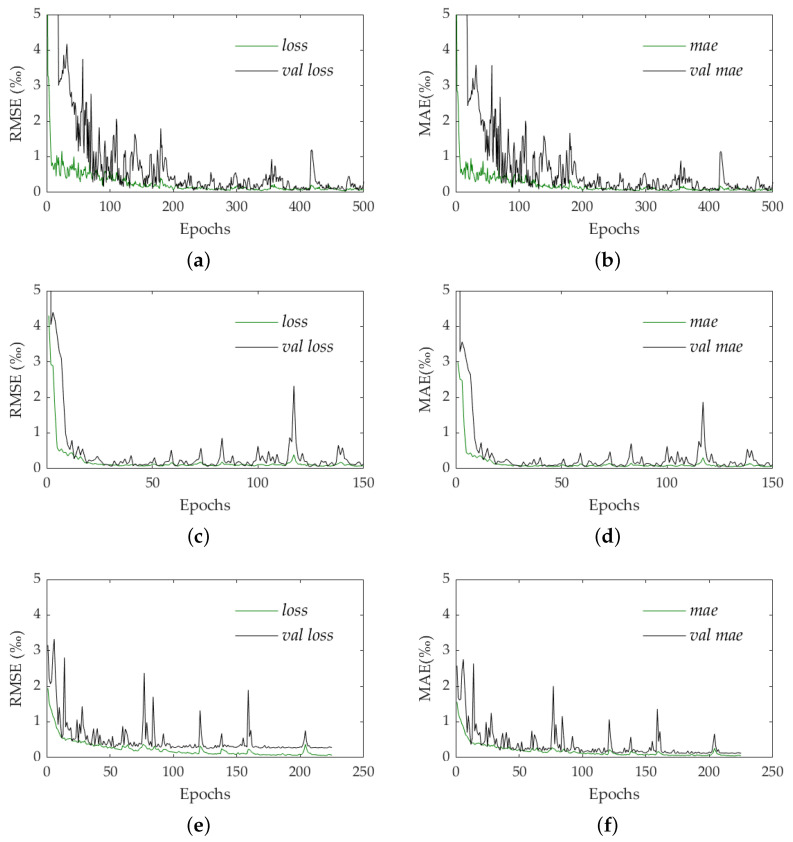
Training datasets validation: (**a**) loss for Training 1; (**b**) MAE for Training 1; (**c**) loss for Training 2; (**d**) MAE for Training 2; (**e**) loss for Training 3; (**f**) MAE for Training 3.

For training validation, the loss function, in terms of RMSE and MAE (Mean Absolute Error), evolution was evaluated in relation to the number of epochs performed for both the training dataset and validation dataset (Figure 7). The RMSE values for the last 50 epochs and for the last epoch are also computed and presented in Table 1 for all three of the training datasets. The average value of the loss in the last 50 epochs was considered as a stopping criterion of the training. For these case studies, this value was 0.1%. All training was computed on Google Colab.

For **Training 1**, 500 epochs were performed, which took approximately 1 h 15. Figure 7a,b show the loss for the training and validation dataset, and the variation of MAE metrics with the number of epochs, respectively. The average value for the last 50 epochs was 0.0760%, with 0.0526% for the last epoch (Table 1). In the case of **Training 2**, 150 epochs were considered, and the training time was approximately 3 h 30 min. Figure 7c,d show the loss and the MAE over the epochs. The average of the last 50 epochs is 0.1092%, with 0.0872% being the last epoch value. **Training 3** requires 250 epochs, and the training time was approximately 9 h. Figure 7e,f show the loss and the MAE over the epochs, with an average of 0.0877% for the last 50 epochs, and 0.0600% in the last epoch. The results show that all three of the training datasets reached metrics that allowed for concluding that the training was carried out successfully.

Table 2 presents the metrics of the analysis performed as defined in Figure 6, namely the RMSE and the MAE values for each case.

**Figure 8:**
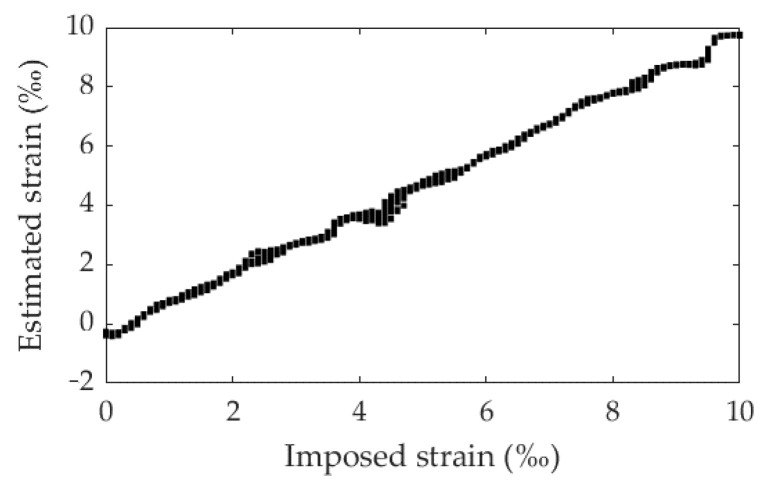
Imposed strain vs. estimated strain: Training 1 and Test D.

To analyse if the model developed is able to estimate strains with values between the values trained, Training 1 (synthetic images between 0% and 10% with an increment of 1%) was tested with Test D (synthetic images between 0% and 10% with an increment of 0.1%). The metrics of Table 2 and Figure 8 clearly demonstrate that the model can estimate intermediate strain values within the range of training dataset values, with an RMSE and MAE of 0.3496% and 0.3190%, respectively.

**Figure 9:**
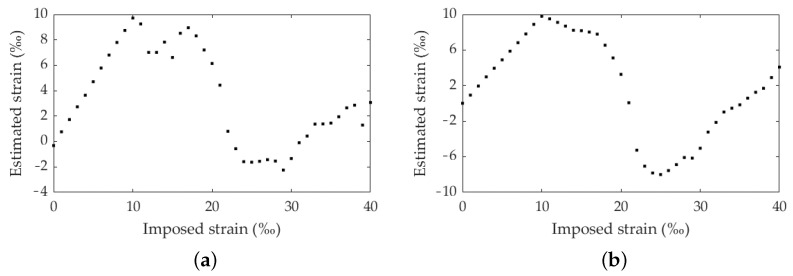
Imposed strain vs. estimated strain: (**a**) Training 1 and Test C; (**b**) Training 2 and Test C.

The analysis of the model ability to estimate strains with higher values than the dataset trained was performed by testing Training 1 and Training 2 (synthetic images between 0% and 10%) with Test C (synthetic images between 0% and 40%). The metrics presented in Table 2 and Figure 9 clearly demonstrate that the model can not estimate strains higher for which the model was trained. All the values estimated from 10% are completely meaningless, as clearly perceptible in the graphics plotted in Figure 9.

The model reaches higher accuracy values for synthetic images. For example, Training 1 and Training 2 have an accuracy lower than 0.35% for Test A, Test B, and Test D (Table 2). However, the best values are always obtained with identical training and test images, namely, Training 1 and Test A, with 0.2941% and 0.2901% for RMSE and MAE, respectively, and for Training 2 and Test D, with 0.0554% and 0.0492% for RMSE and MAE, respectively. For real scenario analysis, Training 3 was tested in Test E, and the values obtained were 0.5702% for RMSE and 0.3597% for MAE, i.e., error values circa 8 to 10 times higher than the results with synthetic images with discretization of 0.1%. On the other hand, the order of magnitude is the same when compared with the analysis with synthetic images with discretization of 1%.

Finally, the capacity of the model trained with synthetic images to estimate the values of the strain in real cases was tested. For these purposes, the Training 1 and Training 2 were tested with Test E. The results reveal errors between 2% and 6.5%, demonstrating that it is not reliable to use a training dataset only composed of synthetic images to estimate strains in real images. However, increasing the discretization level of the trained range values substantially improved the results. This tendency may reveal that the step reduction in the deformation of the training synthetic images may be important for using these training datasets for measuring real cases. However, this requires a significant increase in image resolution.

## 5. Conclusions

The methodology presented in this paper aims for an experimental validation of an image-based architecture for monitoring prestress application in CFRP laminate. The architecture was previously evaluated using synthetic data to benchmark different computer vision algorithms. The best solution, based on the ResNet34 deep learning algorithm with regression, was experimentally tested in a laboratory environment, and the following main conclusions about the model were drawn:

- It allows for measuring intermediate strain levels within the training range. In that sense, the model is able to measure values divisible by 10 between the training values;
- It is not capable of extrapolating for strain levels outside the training range. Thus, it is essential to check the maximum strain to be imposed in real cases, and training the models for higher strain levels;
- For real case scenarios, the error can reach values 10 times higher than using synthetic datasets, i.e, for synthetic datasets, the RMSE value was 0.06% while, for real images, the RMSE value was 0.6%;
- The pre-training with synthetic datasets performed is not able to correctly estimate the strain in real application.

## Figures and Tables

**Table 1:** Metrics for training validation.

Training	RMSE (%)	RMSE (%)
Dataset	Average of Last 50 Epochs	Last Epoch
Training 1	0.0760	0.0526
Training 2	0.1092	0.0872
Training 3	0.0877	0.0600

**Table 2:** Metrics for test datasets (%).

Training	Test A		Test B		Test C		Test D		Test E	
Dataset	RMSE	MAE	RMSE	MAE	RMSE	MAE	RMSE	MAE	RMSE	MAE
Training 1	**0.2941**	**0.2901**	0.3273	0.2935	21.6852	16.6211	0.3496	0.3190	6.5900	6.3671
Training 2	–	–	–	–	23.8490	18.2456	**0.0554**	**0.0492**	2.0126	1.7882
Training 3	–	–	–	–	–	–	9.1181	8.6211	**0.5702**	**0.3597**

## Data Availability

Data are available on request due to privacy or ethical restrictions.

## Abbreviations

CFRP	Carbon-Fiber-Reinforced Polymer
SHM	Structural Health Monitoring
ANN	Artificial Neural Network
CNN	Convolutional Neural Network
RMSE	Root Mean Square Error
MAE	Mean Absolute Error
FOV	Field Of View
